# Evidence for Elevated Cerebrospinal Fluid ERK1/2 Levels in Alzheimer Dementia

**DOI:** 10.4061/2011/739847

**Published:** 2011-11-24

**Authors:** Philipp Spitzer, Heinke Schieb, Heike Kamrowski-Kruck, Markus Otto, Davide Chiasserini, Lucilla Parnetti, Sanna-Kaisa Herukka, Johannes Schuchhardt, Jens Wiltfang, Hans-Wolfgang Klafki

**Affiliations:** ^1^Department of Psychiatry and Psychotherapy, Laboratory for Molecular Neurobiology, LVR-Klinikum Essen, University of Duisburg-Essen, Virchowstraße 174, 45147 Essen, Germany; ^2^Department of Neurology, University of Erlangen-Nuremberg, Schwabachanlage 6, 91054 Erlangen, Germany; ^3^Department of Neurology, University of Ulm, Steinhövelstraße 1, 89075 Ulm, Germany; ^4^Clinica Neurologica, Università di Perugia, Centro Disturbi della Memoria—Unità Valutativa Alzheimer, Ospedale S. Maria della Misericordia, 06156 Perugia, Italy; ^5^Department of Neurology, University of Eastern Finland, Yliopistonranta 1C, 70211 Kuopio, Finland; ^6^MicroDiscovery GmbH, Marienburger Straße 1, 10405 Berlin, Germany

## Abstract

Cerebrospinal fluid (CSF) samples from 33 patients with Alzheimer dementia (AD), 21 patients with mild cognitive impairment who converted to AD during followup (MCI-AD), 25 patients with stable mild cognitive impairment (MCI-stable), and 16 nondemented subjects (ND) were analyzed with a chemiluminescence immunoassay to assess the levels of the mitogen-activated protein kinase ERK1/2 (extracellular signal-regulated kinase 1/2). The results were evaluated in relation to total Tau (tTau), phosphorylated Tau (pTau), and beta-amyloid 42 peptide (A*β*42). CSF-ERK1/2 was significantly increased in the AD group as compared to stable MCI patients and the ND group. Western blot analysis of a pooled cerebrospinal fluid sample revealed that both isoforms, ERK1 and ERK2, and low amounts of doubly phosphorylated ERK2 were detectable. As a predictive diagnostic AD biomarker, CSF-ERK1/2 was inferior to tTau, pTau, and A*β*42.

## 1. Introduction

Alzheimer's disease is a chronic neurodegenerative disorder that causes progressive decline in memory and other higher cortical functions [1]. The underlying neuropathological molecular changes are apparently mirrored by elevated cerebrospinal fluid (CSF) levels of total Tau (tTau) and phosphorylated Tau (pTau) and a reduced concentration of the beta-amyloid 42 peptide (A*β*42). The increase in total Tau is believed to reflect the intensity of the neuronal degeneration, while pTau may indicate the phosphorylation state of the Tau protein (for review, see, e.g., [2]). Biomarkers can complement the clinical disease diagnosis which is currently mainly based on functional symptoms, and they may prove to be helpful in identifying individuals at an early disease stage or at high risk [3, 4]. This will be particularly important once novel disease-modifying therapeutics will be available, which can be expected to produce most benefit when applied early. Biomarkers that reliably report disease-related pathophysiological processes may also be useful to monitor drug efficacy in clinical trials.

An early event in the development of the neurofibrillary changes in Alzheimer's disease is the pathological hyperphosphorylation of Tau [5]. The underlying biochemical mechanisms are poorly understood, and providing deeper insight is a topic of high relevance. Various kinases have been proposed to be possibly implicated, one of which is the MAP kinase ERK2 (reviewed in [6, 7]). In a recent study, we have demonstrated the possibility to detect soluble ERK1/2 in CSF from patients with neuropsychiatric disorders, and we have laid the methodological groundwork for CSF-ERK1/2 quantifications with a 96-well electrochemiluminescence monoplex immunoassay [8]. As a logical extension of this pilot study, we report here the measurement of ERK1/2 in CSF samples from 33 patients with Alzheimer dementia (AD), 21 patients with mild cognitive impairment who converted to AD during followup (MCI-AD), 25 patients with stable mild cognitive impairment (stable MCI), and an age-matched reference cohort (*n* = 16) consisting of nondemented subjects (ND). 

## 2. Materials and Methods 

### 2.1. Patients and Control Subjects

All study procedures were approved by the respective local ethics committees, and the patients or their legal caregivers gave their informed consent. At baseline, a comprehensive medical history was taken, and the patients underwent thorough physical, neurological, and psychiatric examination. For each patient, a computed tomography or an MRI scan was recorded. Alzheimer dementia (AD) was diagnosed according to the NINCDS-ADRDA criteria [9]. Mild cognitive impairment (MCI) was diagnosed, according to the Petersen criteria [10], if a patient had an objective deficiency in one or more cognitive domains (below 1,5 SD compared to an age-, sex-, and education-matched control cohort) but whose general cognitive functioning and whose activities of daily living were not impaired. 

The subjects included in this study were categorized into four groups according to clinical and neuropsychological criteria: (1) patients with Alzheimer dementia (AD, *n* = 33); (2) patients who fulfilled the Petersen criteria for MCI [10] and converted to clinical AD during follow-up (MCI-AD, *n* = 21; average time to conversion 30 months; range: 12–59 months); (3) patients with MCI who remained stable during the follow-up period (MCI-stable, *n* = 25; average follow-up time: 43 months; range: 8–78 months); (4) nondemented individuals referring to the centers for symptoms or other neurological diseases who underwent lumbar puncture for diagnostic reasons (headache, suspected myelopathy, etc.) (ND *n* = 16). The ND group included 4 individuals (“controls”) who were examined for symptoms but were later found to be neurologically healthy and one person who fulfilled the MCI diagnosis at baseline but was later found out to be cognitively healthy. The clinical diagnoses were made blinded to the biomarker results. For the assessment of cognitive dysfunction's severity, the Mini-Mental Status Examination (MMSE) was used [11]. MMSE scores were not available for 7 (out of 16) of the ND subjects who did not show signs of memory decline according to the clinical neurological examination, and for 4 (out of 33) AD patients. As additional neuropsychological testing, MODA (Milan Overall Dementia Assessment [12]) was performed in Perugia. In Kuopio, a set of different neuropsychological tests was applied to specifically test the different cognitive domains. The complete list of tests has already been described elsewhere [13]. Neuropsychological assessment at both sites comprised testing of memory, language, visuospatial skills, attention, orientation, and executive functions. The MODA, which was used in Perugia, furthermore included performance in the activities of daily living as reported by a near relative.

### 2.2. CSF Samples

The CSF samples were collected in Perugia, Italy (*n* = 45) and Kuopio, Finland (*n* = 50). CSF was taken by lumbar puncture and processed according to standardized procedures as described elsewhere [13–15]. After taking CSF for routine tests, a volume of ~10–12 mL of CSF was collected in polypropylene tubes and centrifuged to remove cells. The supernatant (“CSF”) was aliquoted and frozen at −80°C within one hour after sampling. CSF samples containing more than 500 erythrocytes per *μ*L (prior to centrifugation) were excluded from the analysis.

### 2.3. ELISA Measurements of A*β*42, Total Tau, and Phospho-Tau (pT181)

CSF biomarkers were determined by ELISA method (Innotest hTau-Ag, Innotest pTau181-Ag, Innotest *β*-amyloid 1–42, Innogenetics NV, Gent, Belgium). The measurements were performed in the Clinica Neurologica, University of Perugia, Italy and in the Department of Neurology, University of Kuopio, Finland. The tTau, pTau, and A*β*42 data were collected in the context of other studies, and these biomarker results have been published before, at least in parts [13, 16–18]. In the current study, these tTau, pTau, and A*β*42 data were reevaluated in relation to the current ERK1/2 measurements.

### 2.4. ERK1/2 Electrochemiluminescence Monoplex Assay

ERK1/2 in human CSF was measured with the monoplex total ERK1/2 whole cell lysate 96-well electrochemiluminescence assay (Meso Scale Discovery, Gaithersburg, USA) essentially as described previously [8]. For measuring ERK1/2, the CSF samples, which had been received frozen on dry ice and were stored at −80°C, were thawed, aliquoted, and refrozen at −80°C. Immediately prior to the measurements, the CSF aliquots were thawed for a second time, and 111 *μ*L of each sample was mixed with 18.7 *μ*L of an assay buffer concentrate (final concentrations in the assay: 100 mM NaCl, 20 mM Tris-HCl, pH 7.5, 1 mM EDTA, 1 mM EGTA, 1% Triton X-100, plus protease inhibitors, and phosphatase inhibitors (please note that in our previous description of the assay procedure, the final concentration of the added NaCl was erroneously indicated as 150 mM [8]). For the duplicate reads, 50 *μ*L per well was analyzed with the MSD total ERK1/2 monoplex assay according to the manufacturer's instructions. The 1.17-fold dilution resulting from the addition of the assay buffer concentrate was considered for the calculation of the ERK1/2 concentrations. To obtain a suitable reference sample for calculating the standard curves, recombinant activated His-tagged ERK2 (Calbiochem) was initially spiked into a pooled human CSF sample at a concentration of 480 ng/mL (“CSF-stabilized ERK2 stock solution”). Previous observations had indicated that the recombinant-activated kinase was relatively stable under these conditions and could be stored this way in aliquots at −80°C. Starting from this ERK2 stock solution in CSF, serial dilutions (250–16000-fold) were prepared in 1x assay buffer (see above) and measured in triplicates against 1x assay buffer. The standard curve for each assay plate was calculated with the MSD Discovery Workbench 3.0 software. The lower limit of detection (LLOD) was defined as 3 standard deviations (SD) above the blank signal (mean of 3 blanks for each plate). For 9 out of the 95 CSF samples studied here, the ERK1/2 measurements (means of the duplicate reads) were below the corresponding LLOD of the respective assay plate. As a means to include them in the statistical comparison between the diagnostic groups, these samples are reported here with an arbitrary value of 39 pg/mL corresponding to a CSF concentration just below the mean LLOD. When analyzing the correlations between ERK1/2 and age or classical CSF biomarkers, the 9 ERK1/2 measurements below LLOD were excluded. 

### 2.5. Statistical Analysis

Statistical tests were performed with GraphPad Prism 5.01 software (GraphPad Software Inc. La Jolla, Calif, USA). Nonparametric tests (Kruskal-Wallis test followed by Dunn's multiple comparison test and Spearman correlation test) were applied in most cases, since D'Agostino-Pearson omnibus K2 test had indicated that some of the analytes did not follow normal distribution. A level of *P* < 0.05 was regarded as statistically significant. The actual *P* levels are given for descriptive purposes.

### 2.6. Prefractionation of Pooled CSF and Sample Preparation

To facilitate the subsequent Western blot analysis, pooled CSF was prefractionated by isoelectric focusing. Ten individual CSF samples from AD- and non-AD patients were combined. 3.25 mL of the resulting CSF pool was mixed with a 10x protease inhibitor cocktail and a 10x phosphatase inhibitor cocktail (complete Mini and PhosStop, Roche) to yield 1x inhibitor concentrations. Buffer exchange into “off-gel rehydration solution” (7 M urea, 2 M thiourea, 1% (w/v) DTT, 0.5% (v/v) Pharmalyte) and concentration of the sample to a final volume of ~100 *μ*L was achieved with Vivaspin spin concentrators (500 *μ*L, 10 000 MWCO, Sartorius Stedim, Göttingen) according to the manufacturer's protocol. Rehydration solution was added to the concentrated sample to a final volume of 2 mL, and the proteins were separated according to their isoelectric points (pI) on a 3100 off-gel fractionator (Agilent). The isoelectric focusing was performed at a maximum current of 50 *μ*A for 20.000 Vhrs on 13 cm long pH 3–10 IPG strips (GE healthcare). From the resulting 12 fractions, those covering pH ~6.5–~8.5 were pooled. This way, a substantial reduction of albumin was achieved, facilitating the further analysis. The proteins in the prefractionated sample were precipitated with trichloroacetic acid and desoxycholate. Briefly, the sample was mixed with sodium desoxycholate to reach a final concentration of 0.02%. Following 30 min incubation on ice, trichloroacetic acid was added to a final concentration of 15%. After an additional hour on ice, the sample was centrifuged at 16.000 g and 4°C for 30 min. Finally, the pellet was washed twice with ice cold acetone and air dried. For SDS-PAGE, the pellet was dissolved in 60 *μ*L of sample buffer (62.5 mM Tris/HCL pH 6.8, 2% (w/v) SDS, 10% (v/v) Glycerol; 100 mM DTT; 0.005% (w/v) bromophenol blue) for 10 min at 95°C with agitation. 

### 2.7. SDS-PAGE and Western Blotting

The proteins were separated on 12% T/2.7% C SDS-polyacrylamide gels [19] and blotted afterwards on PVDF membranes (Millipore) by semidry transfer at a constant current of 1 mA/cm^2^ for 60 min with 25 mM Tris, 192 mM glycine, and 20% (v/v) methanol [20]. The membranes were blocked in 2% ECL *Advance* blocking agent (GE healthcare) in PBS/0.075% Tween-20 (PBS-T) for 45 min and incubated overnight at 4°C with antibodies diluted in blocking solution. The following antibodies were used: rabbit anti-ERK2 (C-14) sc-154 (Santa Cruz Biotechnology) (working dilution 100 ng/mL); rabbit anti-ERK1/MAPK3 (RnD Systems) (working dilution 250 ng/mL); monoclonal anti-MAP Kinase, activated (clone MAPK-YT) (Sigma) (working dilution 1 : 1000). The next day the membranes were washed with PBS-T and incubated for 1 h at room temperature with horseradish-peroxidase-coupled secondary antibodies. After washing with PBS-T, the blots were developed with ECL-plus chemiluminescent substrate according to the manufacturer's instructions (GE Healthcare) and visualized with an Intas Imager (Intas, Göttingen, Germany).

## 3. Results

### 3.1. Diagnostic Groups, Demographical Data, MMSE Scores, and CSF Biomarkers

A summary of the demographical data of the 4 diagnostic groups under investigation, as well as mean MMSE scores and median levels of the CSF biomarkers tTau, pTau, and A*β*42is shown in Table 1. ANOVA test indicated that the mean ages did not differ significantly between the 4 groups (*P* = 0.0999). The CSF levels (rank sums) of tTau and pTau were significantly higher in the AD- and the MCI-AD groups than in the MCI-stable and ND groups (Kruskal-Wallis test followed by Dunn's multiple comparison test). A*β*42 was significantly lower in AD- and MCI-AD patients than in MCI-stable and ND subjects (supplementary information, see Figure S1 in Supplementary Material available online at doi:10.4061/2011/739847). 

### 3.2. Quantification of ERK1/2 in CSF

The ERK1/2 concentrations in the CSF samples were quantified with the total ERK1/2 monoplex electrochemiluminescence assay. The standard curves were calculated from measurements of serial dilutions in assay buffer of a “CSF-stabilized” ERK2 stock solution (see Materials and Methods). The lower limit of detection of the assay (LLOD) was defined as 3 standard deviations (SD) above the blank signal. For the 4 assay plates employed here, the LLODs were calculated as 31.1 pg/mL, 31.4 pg/mL, 40.5 pg/mL, and 31.7 pg/mL, respectively (mean LLOD ± SD: 33.7 ± 4.6 pg/mL). Taking into account the 1.17-fold dilution of the CSF samples resulting from addition of an assay buffer concentrate prior to the measurements, the mean LLOD of the assay corresponded to an ERK1/2 concentration of 39.4 pg/mL in undiluted CSF. In total, for this study, 95 CSF samples were included in the analysis. With regard to the raw signal counts, the coefficient of variation (CV) between the duplicate reads was on average 2.4% ±2 (mean ± SD). The maximum CV between the respective duplicate reads (raw signal counts) observed in the whole sample was 10.3%, indicating an acceptable technical assay performance. It should be noted, however, that the imprecision regarding the individually calculated CSF-ERK1/2 concentrations for the duplicates was higher (CV ± SD: 8.5% ±7.5%, *n* = 86; the measurements with mean calculated ERK1/2 levels below the LLOD were excluded).

### 3.3. CSF-ERK1/2 Levels in the 4 Diagnostic Groups

**Figure 1:**
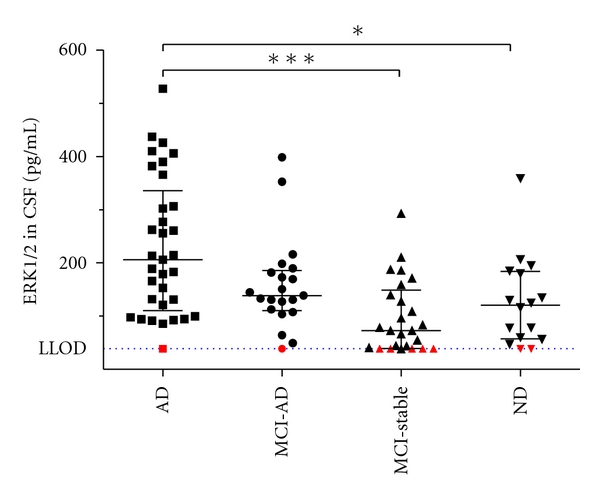
Scatterplot showing CSF levels of ERK1/2 in the 4 diagnostic groups. The bars indicate medians and the interquartile ranges. The red symbols indicate those 9 samples with signals below LLOD that are reported here arbitrarily as 39 pg/mL. The differences in rank sums between the AD and the ND groups and between AD and MCI-stable were statistically significant: *P < 0.05, ***P < 0.001 (Kruskal-Wallis test followed by Dunn's multiple comparison test). LLOD: mean lower limit of detection of ERK in undiluted CSF (39.4 pg/mL).

For comparing the CSF-ERK1/2 levels between the 4 diagnostic groups, those 9 samples with CSF-ERK1/2 measurements (means from duplicate reads) below the respective assay plate LLOD are reported here with an artificially set value of 39 pg/mL. This corresponds to a CSF concentration just below the mean LLOD (see above). As a group, the AD patients showed significantly higher CSF-ERK1/2 concentrations than the nondemented individuals (*P* < 0.05) and the stable MCI cohort (*P* < 0.001, Kruskal-Wallis test followed by Dunn's multiple comparison test) (Figure 1). The median ERK1/2 concentrations in the MCI-AD group were in between those of the AD patients and the stable MCI/ND cohort. However, as shown in Figure 1, we observed a clear overlap between groups. As a consequence, the group comparisons between MCI-AD and MCI-stable, MCI-AD and AD, MCI-AD and ND, and MCI-stable and ND did not indicate statistically significant differences (Kruskal-Wallis test followed by Dunn's multiple comparison test). To additionally evaluate whether the large overlap between the MCI-stable and MCI-AD groups might be attributed to those MCI-stable subjects in our sample with relatively short follow-up times, we repeated the calculations with predefined minimum follow-up times for MCI-stable of 24 and 36 months, respectively. Again, only the group differences between AD versus MCI-stable and AD versus ND reached statistical significance (Kruskal-Wallis test with Dunn's multiple comparison posttest) (data not shown). 

**Figure 2:**
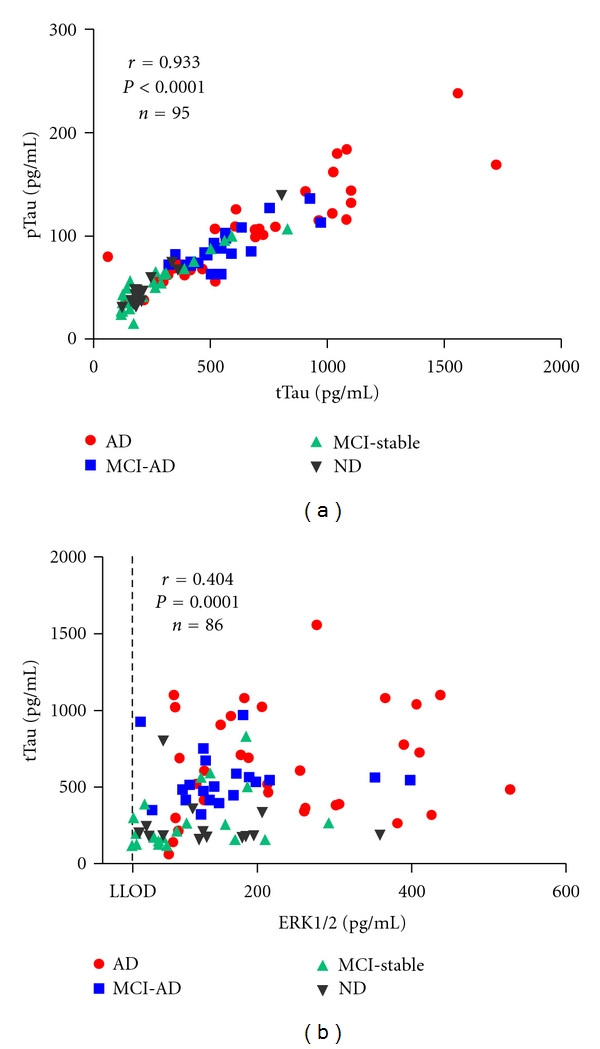
Correlations between tTau and pTau and between tTau and ERK1/2. In the whole sample, the CSF concentrations of tTau were significantly correlated with (a) pTau and (b) ERK1/2. ERK1/2 measurements below the LLOD of the respective assay plate (*n* = 9) were excluded from the Spearman correlation test.

To investigate the possible diagnostic added value of combining CSF-ERK1/2 levels with classical CSF biomarkers, we compared our current results with the CSF concentrations of tTau, pTau, and A*β*42. The CSF concentrations of pTau and tTau were strongly correlated with each other (Spearman test) (Figure 2(a)). A significant correlation between ERK1/2 and tTau and between ERK1/2 and pTau was observed, when considering the whole sample (Figure 2(b)), while such a correlation did not reach the statistical significance in any diagnostic group. The CSF ERK1/2 levels were negatively correlated with A*β*42 in the whole sample and in the AD group. A summary of nonparametric Spearman correlation analysis for ERK1/2 versus tTau, pTau, and A*β*42 is shown in Table 2. 

The measurements of the CSF biomarkers tTau, pTau, and A*β*42 are known to show a substantial degree of variation between different centers [21]. When we performed separate correlation analyses for the 2 centers involved in this study, significant negative correlations were observed between ERK1/2 and A*β*42 in the samples from both sites when no classification into diagnostic groups was applied, and in the AD group from Kuopio. ERK1/2 and tTau as well as ERK1/2 and pTau were significantly correlated with each other in the samples from Perugia when all subjects were considered, but not in any of the diagnostic groups per se (supplementary information, Table S1).

### 3.4. Correlation of CSF-ERK1/2 with MMSE

**Figure 3:**
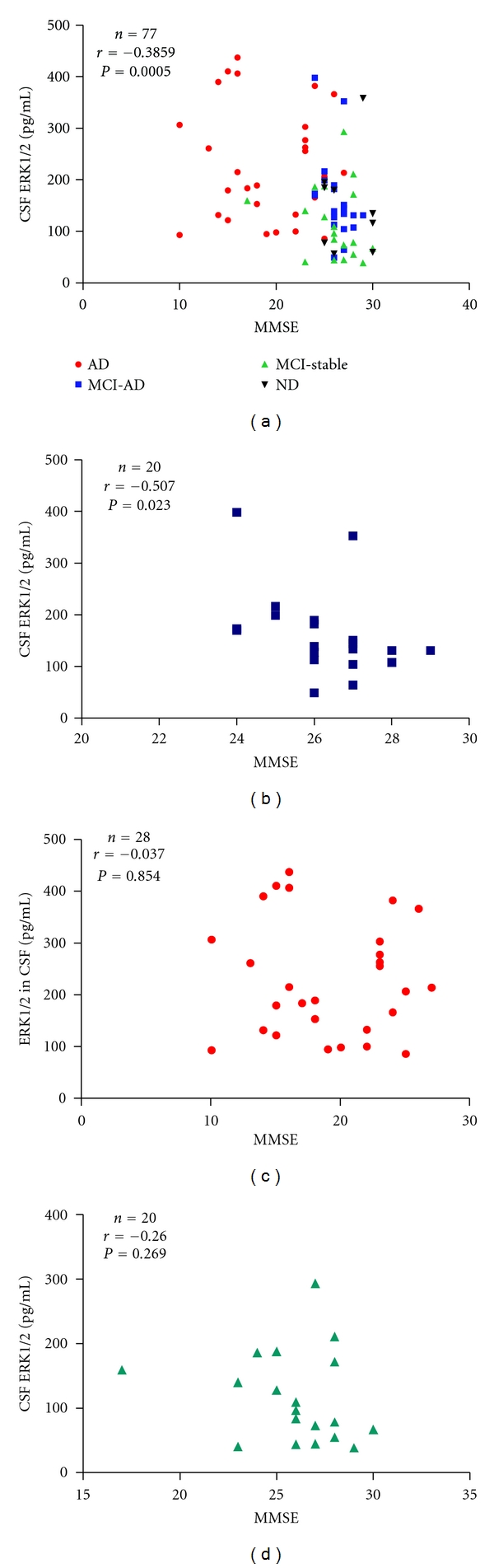
Scatterplots of MMSE scores plotted against CSF-ERK1/2 levels. (a) Whole sample (*n* = 77). (b) MCI-AD group (*n* = 20). (c) AD patients (*n* = 28). (d) MCI-stable (*n* = 20). The Spearman correlation coefficients (*r*) and the two-tailed *P* values are indicated. Those samples with ERK1/2 measurements below LLOD were excluded from the analysis.

For 84 of the subjects included in this study (AD: *n* = 29, MCI-AD: *n* = 21, MCI-stable: *n* = 25, ND: *n* = 9), MMSE scores as a measure for cognitive functions were available. 77 out of these with calculated CSF-ERK1/2 concentrations above the LLOD were subjected to correlation analysis. In the whole sample, CSF-ERK1/2 showed a statistically significant negative Spearman correlation with the MMSE scores (Figure 3(a)). When the AD-, MCI-AD-, and MCI-stable groups were analyzed individually, a significant negative Spearman correlation between MMSE scores and CSF-ERK1/2 was observed only in the MCI-AD group (Figure 3(b)) but not in the AD group or the stable MCI patients (Figures 3(c) and 3(d)).

### 3.5. Receiver-Operator Characteristic Curves

**Figure 4:**
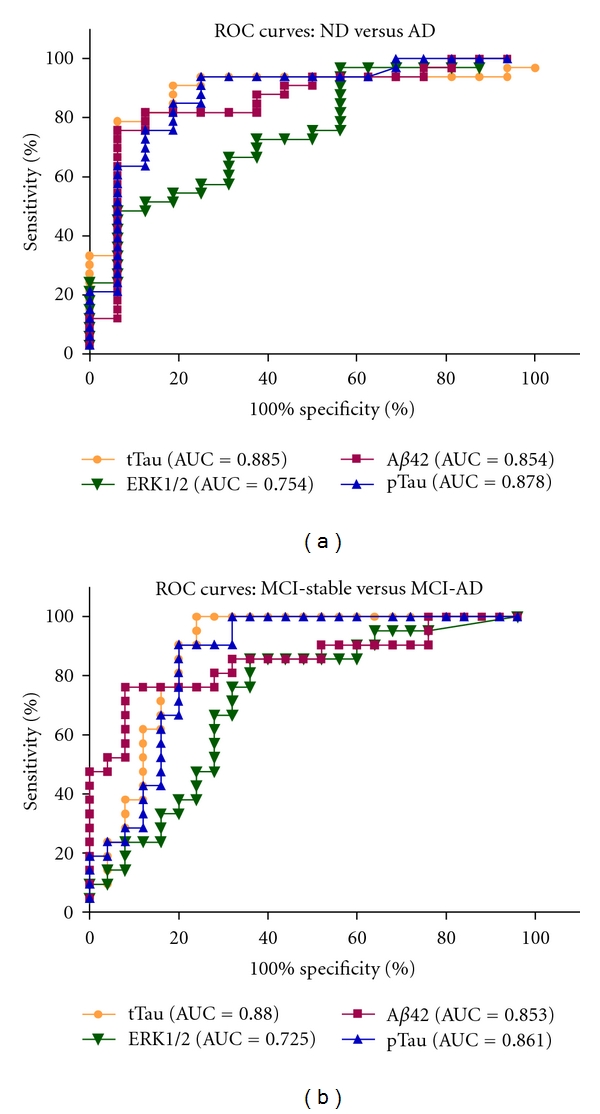
ROC curves for CSF-ERK1/2 levels and classical biomarkers: (a) ROC curves for A*β*42, tTau, pTau, and ERK1/2 (at baseline) for the discrimination between ND and AD. (b) ROC curves for A*β*42, tTau, pTau, and ERK1/2 (at baseline) for the discrimination between MCI-stable and MCI-AD. AUC: area under the curve. ERK1/2 measurements below LLOD are reported as 39 pg/mL in (a) and (b).

To evaluate the possibility to use CSF-ERK1/2 levels for the discrimination between relevant diagnostic groups, receiver-operator characteristic curves (ROC curves) were calculated. Figure 4(a) shows the ROC curves for ERK1/2, tTau, pTau, and A*β*42 for the discrimination of AD from ND, Figure 4(b) for the discrimination of MCI-AD from stable MCI. The calculated areas under the curves (AUCs) indicate that as single biomarkers tTau, pTau, and A*β*42 were clearly superior to CSF-ERK1/2.

### 3.6. Immunoblot Analysis of Prefractionated CSF

**Figure 5:**
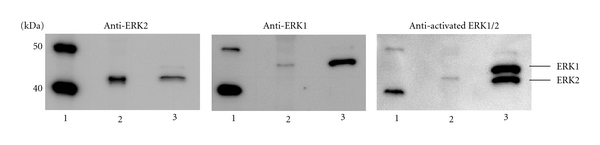
Detection of ERK1, ERK2, and doubly phosphorylated ERK2 in a pooled human CSF sample. Following prefractionation of 3.25 mL of pooled CSF on an Off-gel fractionator, the proteins with isoelectric points in the pH range from ~6.5 to ~8.5 were pooled, precipitated with trichloroacetic acid, and analyzed by SDS-PAGE/Western blot with the indicated antibodies. A sample corresponding to ~0.54 mL of the original CSF-pool was loaded on each lane. (1) Magic mark protein standard, (2) CSF pool, (3) positive control (cell lysate of okadaic-acid-treated SH-SY5Y cells).

To provide additional information regarding the ERK1/2 isoform(s) contributing to the signals detected by the ERK1/2 electrochemiluminescence assay, a pooled CSF sample was prefractionated by off-gel isoelectric focusing to facilitate the subsequent Western blot analysis. The fractions covering the pH range from ~6.5 to ~8.5 were combined and separated by SDS-polyacrylamide-gel electrophoresis followed by Western blotting. Probing with a panel of anti-ERK antibodies revealed the presence of ERK1, ERK2, and doubly phosphorylated ERK2 in the pooled human CSF (Figure 5). Visual comparison of the band intensities in pooled CSF with those obtained with a lysate from SH-SY5Y cells loaded as a positive control suggested that ERK2 was more abundant than ERK1.

## 4. Discussion

In the present study, we have confirmed our previous finding of measurable levels of the MAP kinase ERK1/2 in human CSF [8], and we have applied the methods established in that previous work to evaluate the CSF-ERK1/2 concentrations in an age-matched sample including AD patients, MCI patients who converted to AD during followup (MCI-AD), MCI patients who remained stable, and a nondemented reference cohort. As a group, the AD patients showed significantly higher CSF-ERK1/2 levels than the MCI-stable group and the nondemented reference cohort. The individual ERK1/2 concentrations in the CSF samples displayed substantial overlap between the diagnostic groups, and as a single biomarker, CSF-ERK1/2 was clearly inferior to the classical AD biomarkers tTau, pTau, and A*β*42, as is obvious from the ROC curves shown in Figure 4.

In line with published biomarker studies [4, 22], in our sample, A*β*42, tTau and pTau, in CSF were significantly altered at baseline in those MCI patients who later converted to AD (supplementary information, Figure S1). This was not the case for CSF-ERK1/2. In the AD group, but not in the MCI-AD patients, CSF-ERK1/2 was statistically significantly elevated as compared to the ND and MCI-stable groups. Therefore, it appears that the increase in CSF-ERK1/2 may possibly occur at a later disease stage than those of tTau and pTau. In the whole sample, CSF-ERK1/2 was significantly correlated with the CSF concentrations of tTau and pTau and negatively correlated with A*β*42. A correlation between ERK1/2 and tTau had also been observed in our previous study in a small set of CSF samples from patients with different neuropsychiatric disorders [8]. A simple explanation could be that both proteins are released in parallel when neurons degenerate. However, the observed correlation between ERK1/2 and tau in the whole sample in our present study was not particularly strong (Spearman *r* = 0.404 as compared to *r* = 0.933 for the correlation between pTau and tTau), and the association between the two did not reach statistical significance in any of the diagnostic groups per se. To gain more insight whether the increase in CSF-ERK1/2 in AD represents simply another marker of neuronal destruction in addition to Tau, or if possibly different cell populations or distinct underlying molecular mechanisms are reflected, it might be interesting to measure CSF-ERK1/2 levels in patients with other non-AD neurodegenerative disorders. In a recent study, a statistically significant correlation between the CSF levels of tTau and ERK1/2 was not observed in patients with Creutzfeldt-Jakob disease [23]. Interestingly, in our current study, we found a negative association of baseline CSF-ERK1/2 and the MMSE scores in the MCI-AD group. Provided these findings can be confirmed in a larger sample, and possibly also with other measures of cognitive function, we believe that our preliminary results suggest that the change in CSF-ERK1/2 occurs later than those of Tau and A*β*42. One can therefore speculate that in the long run, CSF-ERK1/2 may turn out to be of interest as a biomarker sensitive to disease progression at the MCI stage. Whether or not the ApoE genotype has an impact on the CSF-ERK1/2 levels has to remain open at this stage since a larger sample size will be required for reasonable statistical evaluation. 

The mitogen-activated protein/extracellular signal-regulated kinases ERK1 and ERK2 are ubiquitous components of cellular signal transduction pathways regulating diverse downstream processes, including proliferation, differentiation, survival, and apoptosis [24, 25]. In the adult brain, ERKs are abundantly expressed and are involved in the regulation of activity-dependent neuronal functions, such as synaptic plasticity, long-term potentiation, long-term depression, and cell survival (for reviews, see [26–28]). All three mitogen-activated kinase (MAPK) pathways, ERK, JNK and p38 pathways, were reported to be activated in vulnerable neurons in AD brains and were proposed to play an important role in the pathogenesis [29]. In agreement with the observations in CSF from Creutzfeldt-Jakob disease patients [23], we demonstrate herein the presence of both isoforms, ERK1 and ERK2, in a pooled CSF sample. In rat brains, differences in the regional distributions of these 2 ERK isoforms were reported [30]. We have furthermore confirmed our previous finding of detectable amounts of the activated (i.e., doubly phosphorylated) form of ERK2 in pooled CSF. To gain further insight into pathogenic and pathophysiological molecular changes, it might be particularly interesting to address in future studies the activation state of ERK1/2 in CSF from AD patients. An essential prerequisite for this kind of study, however, will be the availability of a highly sensitive and reliable assay for the measurement of doubly phosphorylated ERK1/2. 

Taken together, we report here a statistically significant elevation of CSF-ERK1/2 in AD patients as compared to ND and MCI-stable subjects. As a predictive diagnostic tool, CSF-ERK1/2 turned out to be inferior to the “classical” CSF biomarkers tTau, pTau, and A*β*42. Although our findings need to be confirmed and extended in future studies, the observations suggest that the increase in ERK-1/2 levels may occur at a later stage during disease development or progression than the changes of tTau, pTau, and A*β*42 in CSF.

## Supplementary Material

The CSF levels of the classical biomarkers tTau, pTau, and A*β*42 and the ages of the subjects included in the 4 diagnostic groups under investigation were analyzed by Kruskal-Wallis test followed by Dunn's multiple comparison test (Figure S1). The CSF concentrations of tTau and pTau were significantly higher in the AD and MCI-AD patients than in the MCIstable and ND groups. CSF A*β*42 was significantly lower in AD and MCI-AD than in MCIstable and ND. Significant group differences in the median or mean ages were not observed with Kruskal-Wallis and ANOVA tests.
To complement the analysis for correlations between CSF-ERK1/2 and classical CSF biomarkers in the whole sample and to address the possibility of center effects, additional separate correlation analyses were done for each of the two centers involved. The results are summarized in Table S1. CSF-ERK1/2 and A*β*42 were inversely correlated in the samples from both sides when no classification into diagnostic groups was applied. Furthermore, a statistically significant negative association between ERK1/2 and A*β*42 was observed in the AD group from Kuopio. The CSF-levels of tTau and of pTau were significantly correlated with CSF-ERK1/2 in the Perugia sample when no classification into diagnostic groups was applied.

## Figures and Tables

**Table 1:** Demographical data, MMSE scores, and classical CSF biomarkers*.

		AD	MCI-AD	MCI-stable	ND
	*n*	33	21	25	16
Age (years)	Mean	68.4	66	69	63.3
	SD	7.6	8.5	7.4	8.4

Gender	m/f	15/18	7/14	10/15	6/10

MMSE	Mean	19 (*n* = 29)	26.3	26	27.3 (*n* = 9)
	SD	4.8	1.3	2.5	2.3

tTau (pg/mL)	Median	609.8	536.5	210	185.5
	25%–75% percentile	372.6–1023	432.2–611.5	145–349.4	176.9–236.6

pTau (pg/mL)	Median	106	84	50	45.2
	25%–75% percentile	67.5–129.0	73.1–100.3	35.0–66.6	37.2–57.3

A*β*42 (pg/mL)	Median	432	397.2	745	794.5
	25%–75% percentile	361.5–523.6	344.8–553.2	589.3–981.5	635.8–844.8

*The CSF levels of tTau, pTau, and A*β*42 are shown here for comparative purposes. The actual measurements were performed within the context of other studies, and some of the data have been published before [13, 16–18].

**Table 2:** Analysis for correlations between ERK1/2 and classical biomarkers*.

		ERK1/2		
Patients group	CSF marker	*r*	*P* value	*P* value summary
Whole sample (*n* = 86)^#^	A*β*_1-42_	−0.436	< 0.0001	Significant
	t-Tau	0.404	0.0001	Significant
	p-Tau	0.384	0.0003	Significant
	Age	0.0001	0.999	n.s.

ND (*n* = 14)^#^	A*β*_1-42_	−0.0242	0.9346	n.s.
	t-Tau	−0.174	0.5528	n.s.
	p-Tau	−0.0022	0.9941	n.s.
	Age	0.0726	0.8052	n.s.

MCI-stable (*n* = 20)^#^	A*β*_1-42_	0.0947	0.6912	n.s.
	t-Tau	0.399	0.0816	n.s.
	p-Tau	0.433	0.0563	n.s.
	Age	0.0845	0.7231	n.s.

MCI-AD (*n* = 20)^#^	A*β*_1-42_	−0.0992	0.6772	n.s.
	t-Tau	0.325	0.1623	n.s.
	p-Tau	0.0248	0.9173	n.s.
	Age	0.171	0.4723	n.s.

AD (*n* = 32)^#^	A*β*_1-42_	−0.544	0.0013	Significant
	t-Tau	0.176	0.3343	n.s.
	p-Tau	0.161	0.3789	n.s.
	Age	−0.136	0.457	n.s.

*Spearman correlation coefficients (*r*) and *P* values (two tailed) are indicated.

^#^ERK1/2 measurements below LLOD (*n* = 9) were excluded from the correlation analysis.
