# Supplementary material for: Evidence for Elevated Cerebrospinal Fluid ERK1/2 Levels in Alzheimer Dementia
Source: Int J Alzheimers Dis. 2011 Nov 24;2011:739847. doi: 10.4061/2011/739847 (PMC3227514; doi:10.4061/2011/739847)
Supplement: Supplementary file 1 — The CSF levels of the classical biomarkers tTau, pTau, and Aβ42 and the ages of the subjects included in the 4 diagnostic groups under investigation were analyzed by Kruskal-Wallis test followed by Dunn's multiple comparison test (Figure S1). The CSF concentrations of tTau and pTau were significantly higher in the AD and MCI-AD patients than in the MCIstable and ND groups. CSF Aβ42 was significantly lower in AD and MCI-AD than in MCIstable and ND. Significant group differences in the median or mean ages were not observed with Kruskal-Wallis and ANOVA tests. To complement the analysis for correlations between CSF-ERK1/2 and classical CSF biomarkers in the whole sample and to address the possibility of center effects, additional separate correlation analyses were done for each of the two centers involved. The results are summarized in Table S1. CSF-ERK1/2 and Aβ42 were inversely correlated in the samples from both sides when no classification into diagnostic groups was applied. Furthermore, a statistically significant negative association between ERK1/2 and Aβ42 was observed in the AD group from Kuopio. The CSF-levels of tTau and of pTau were significantly correlated with CSF-ERK1/2 in the Perugia sample when no classification into diagnostic groups was applied. [file 739847.f1.pdf]

Supplementary information to:

Evidence for Elevated Cerebrospinal Fluid ERK1/2 Levels in Alzheimer's Disease

Philipp Spitzer, Heinke Schieb, Heike Kamrowski-Kruck, Markus Otto, Davide Chiasserini, Lucilla Parnetti, Sanna-Kaisa Herukka, Johannes Schuchhardt, Jens Wiltfang, Hans-Wolfgang Klafki

1) Age and CSF levels of classical biomarkers tTau, pTau, and Aβ42 in the 4 diagnostic groups

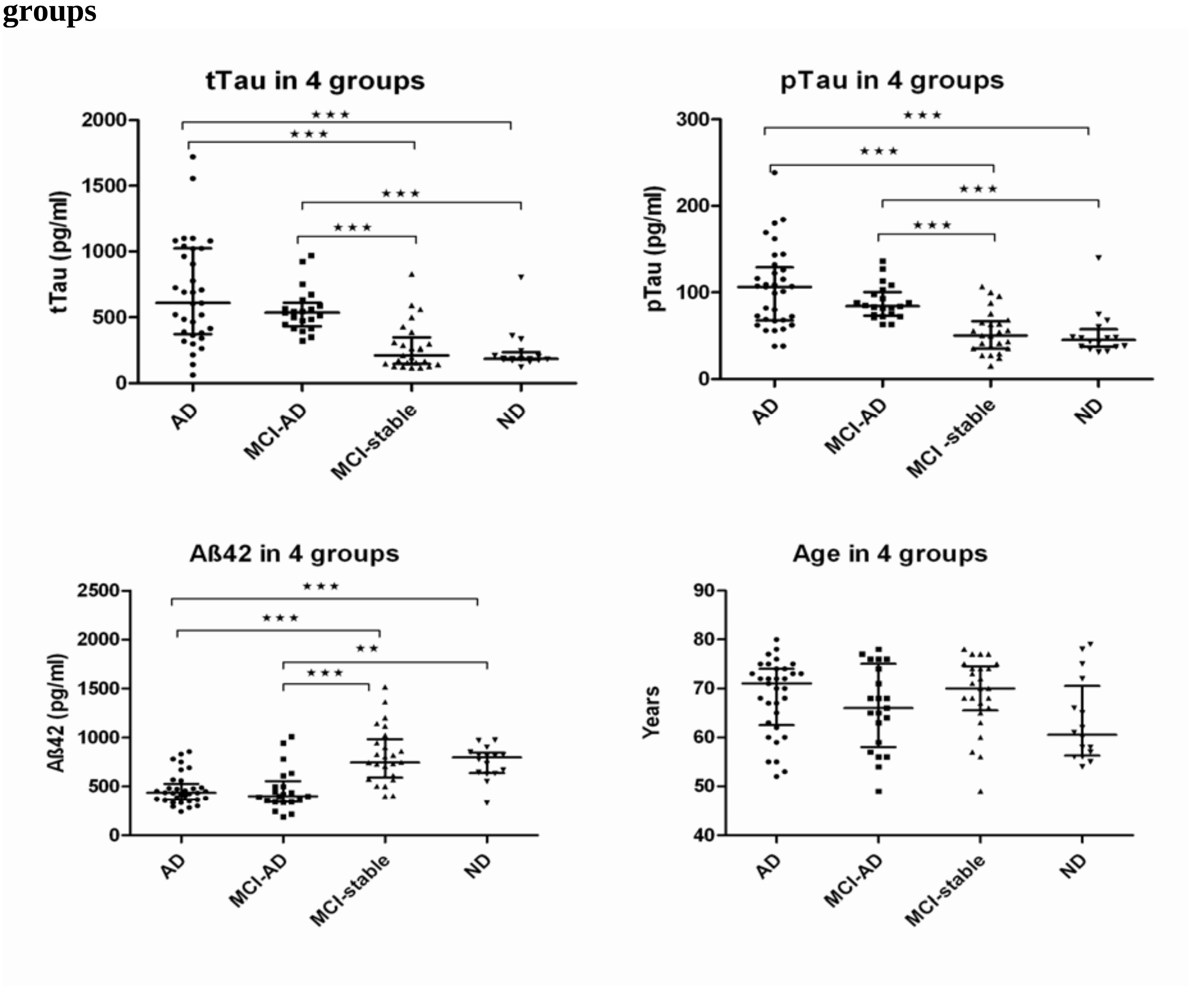

Figure S1: Age distribution and CSF levels of classical biomarkers tTau, pTau, and Aβ42: Non-parametric Kruskal-Wallis test followed by Dunn's multiple comparison test indicated significantly higher tTau and pTau levels (rank sums) in AD- and MCI-AD patients than in the MCI-stable and ND groups. CSF-Aβ42 (rank sums) was significantly lower in the AD and MCI-AD groups than in MCI-stable and ND groups (*: $p < 0.05$; **: $p < 0.01$; ***: $p < 0.001$). Neither median nor mean ages differed significantly between the 4 groups ($p = 0.1366$ with Kruskal-Wallis test and $p = 0.0999$ with parametric ANOVA test). The bars indicate medians and interquartile ranges. The CSF levels of tTau, pTau and Aβ42 are shown here for comparative purposes. The actual measurements were performed within the context of other studies, and some of the data have been published before [1-4].

2) Table S1: Analysis for correlations between CSF-ERK1/2 and classical biomarkers for each center*.

		tTau	pTau	Aβ42	Age
**Perugia all (n=41)**	Spearman r<br>p-value	0.603<br><0.0001	0.539<br>0.0003	-0.343<br>0.028	0.081<br>0.613
**Perugia AD (n=15)**	Spearman r<br>p-value	0.359<br>0.189	0.271<br>0.328	-0.45<br>0.092	-0.12<br>0.670
**Perugia MCI-AD (n=11)**	Spearman r<br>p-value	0.436<br>0.18	-0.319<br>0.339	-0.055<br>0.873	0.083<br>0.809
**Perugia MCI-stable (n=13)**	Spearman r<br>p-value	0.460<br>0.114	0.460<br>0.114	0.363<br>0.223	0.036<br>0.907
**Perugia ND (n=2)**		n too small	n too small	n too small	n too small
**Kuopio all (n=45)**	Spearman r<br>p-value	0.245<br>0.105	0.244<br>0.107	-0.520<br>0.0002	-0.178<br>0.242
**Kuopio AD (n=17)**	Spearman r<br>p-value	0.123<br>0.639	0.100<br>0.701	-0.544<br>0.024	0.058<br>0.825
**Kuopio MCI-AD (n=9)**	Spearman r<br>p-value	0.067<br>0.880	0.250<br>0.521	-0.150<br>0.708	0.159<br>0.678
**Kuopio MCI-stable (n=7)**	Spearman r<br>p-value	0.357<br>0.444	0.357<br>0.444	-0.071<br>0.906	-0.487<br>0.267
**Kuopio ND (n=12)**	Spearman r<br>p-value	-0.196<br>0.542	-0.357<br>0.255	0.098<br>0.762	-0.158<br>0.625

* Spearman correlation coefficients (r) and p-values (two-tailed) are indicated.
ERK1/2 measurements below LLOD (n = 9) were excluded from the correlation analysis.

References cited in the supplementary information:

[1] Herukka SK, Helisalmi S, Hallikainen M, Tervo S, Soininen H, Pirttila T (2007) CSF Abeta42, Tau and phosphorylated Tau, APOE epsilon4 allele and MCI type in progressive MCI. *Neurobiol Aging* **28**, 507-514.

[2] Lanari A, Parnetti L (2009) Cerebrospinal fluid biomarkers and prediction of conversion in patients with mild cognitive impairment: 4-year follow-up in a routine clinical setting. *ScientificWorldJournal* **9**, 961-966.

[3] Parnetti L, Lanari A, Silvestrelli G, Saggese E, Reboldi P (2006) Diagnosing prodromal Alzheimer's disease: role of CSF biochemical markers. *Mech Ageing Dev* **127**, 129-132.
[4] Tapiola T, Alafuzoff I, Herukka SK, Parkkinen L, Hartikainen P, Soininen H, Pirttila T (2009) Cerebrospinal fluid {beta}-amyloid 42 and tau proteins as biomarkers of Alzheimer-type pathologic changes in the brain. *Arch Neurol* **66**, 382-389.
